# Growth Disorder Gene Plays a Big Role in Normal Size Variation

**DOI:** 10.1371/journal.pbio.0030163

**Published:** 2005-04-05

The presence of a small number of discrete forms—as you find in a classic Mendelian trait like eye color—suggests that the phenotype is controlled by a very small number of genes. In contrast, a complex trait such as body size is influenced by multiple genes as well as environmental factors, giving rise to a continuous spectrum of phenotypes. This causal complexity makes discovery of the genetic determinants of the trait—so-called quantitative trait loci (QTLs)—very difficult. In this issue, Fiona Oliver, Julian Christians, and colleagues extend their work on a single QTL with a large effect on body size variation in mice, and show that the responsible gene is one already linked to a Mendelian growth disorder in humans.

In previous work, mice were divergently selected for large or small body size, revealing a QTL on the X chromosome that causes a 20% difference in growth rate. In this study, the authors further refined the map of the area to 660 kilobases, and found it contains only four genes: glypican-3 and glypican-4 (Gpc3 and Gpc4), and two other genes of unknown function. The glypicans are membrane-bound growth regulators; loss of function of Gpc3 in humans causes a rare syndrome of overgrowth, skeletal and other abnormalities, and neonatal death. Since none of the four genes showed coding-region variations that might explain the differences between the large and small mice, the authors examined expression levels. In Gpc3, but not the others, size correlated with a significant difference in gene expression: larger mice had low levels of the messenger RNA, and smaller mice had high levels&mdash;the same pattern seen in the human loss-of-function disorder. The authors identified several non-coding polymorphisms in Gpc3 that differed between the two forms, although it remains to be seen whether the differential effect on growth is due to these or other DNA differences nearby.[Fig pbio-0030163-g001]

**Figure pbio-0030163-g001:**
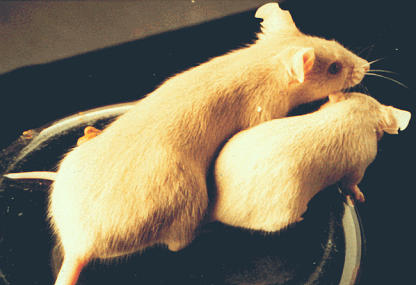
Variation in the expression of a protein involved in a rare human overgrowth syndrome contributes to size variation in mice (Photo: Lutz B&uuml;nger, Scottish Agricultural College)

The results from this study point out an important feature of inheritance, namely, that a gene implicated in a Mendelian trait can also contribute to quantitative variation. While catastrophic expression failure, such as a loss of function, can cause disease, smaller changes in expression of the same gene may simply help fill out the bell curve of normal variation.

